# Supplementary material for: The interactome of intact mitochondria by cross-linking mass spectrometry provides evidence for coexisting respiratory supercomplexes
Source: Mol Cell Proteomics. 2017 Dec 8;17(2):216–32. doi: 10.1074/mcp.RA117.000470 (PMC5795388; doi:10.1074/mcp.RA117.000470)
Supplement: Supplemental Data [file supp_RA117.000470_133922_0_supp_23978_fzffwf.zip › spectra_annotation/mito_spectra_annotation/87-2-4-1-2-1.pdf]

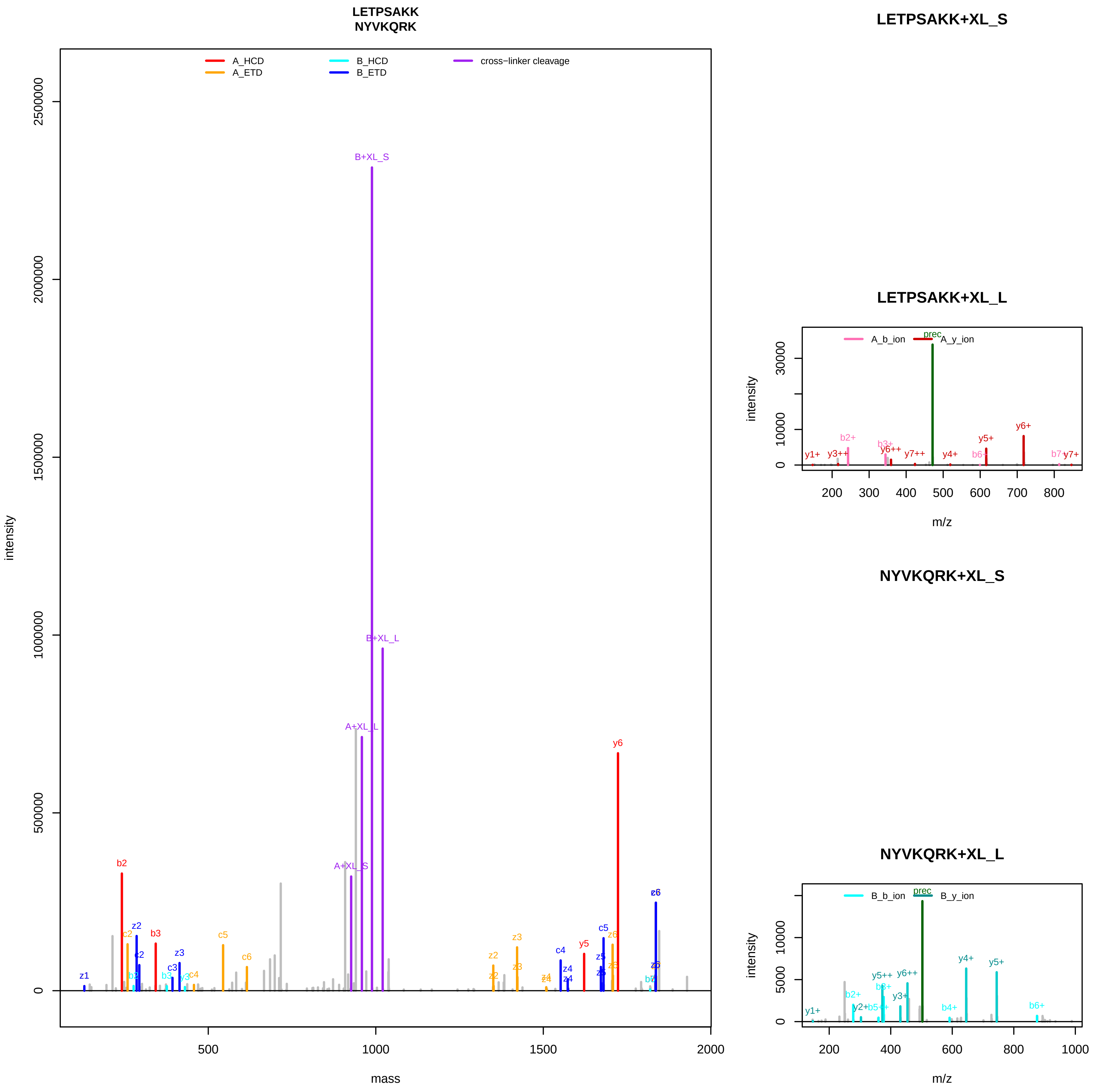

LETPSAKK
NYVKQRK
A_HCD
A_ETD
B_HCD
B_ETD
cross−linker cleavage
B+XL_S
B+XL_L
A+XL_L
A+XL_S
y6
b2
b3
c2
z2
c3
z3
b3
y3
c4
c5
c6
z2
z3
z4
c4
z4
y5
c5
z5
z6
z6
b7
intensity
mass
0
500000
1000000
1500000
2000000
2500000
500
1000
1500
2000
LETPSAKK+XL_S
LETPSAKK+XL_L
A_b_ion
A_y_ion
prec
y1+
y3++
b2+
b3+
y6++
y7++
y4+
b6+
y5+
y6+
b7+
y7+
intensity
m/z
0
10000
30000
200
300
400
500
600
700
800
NYVKQRK+XL_S
NYVKQRK+XL_L
B_b_ion
B_y_ion
prec
y1+
b2+
y2+
b5++
b3+
y5++
y3+
y6++
b4+
y4+
y5+
b6+
intensity
m/z
0
5000
10000
200
400
600
800
1000
